# P75 Mechanisms of increased resistance to chlorhexidine in *Klebsiella pneumoniae*

**DOI:** 10.1093/jacamr/dlaf230.082

**Published:** 2025-12-04

**Authors:** Yige Sima, Isobel Garratt, Tiffany Taylor, Matthew Wand, Mark Sutton, Eshwar Mahenthiralingam, Brian V Jones

**Affiliations:** University of Bath, Bath, UK; University of Bath, Bath, UK; University of Bath, Bath, UK; UK Health Security Agency, Salisbury, UK; UK Health Security Agency, Salisbury, UK; Cardiff University, Cardiff, UK; University of Bath, Bath, UK

## Abstract

**Objectives:**

Biocides are important antimicrobial agents used extensively in healthcare settings as antiseptics or disinfectants. The increasing use of biocides has been driven by efforts to reduce antibiotic use and the Covid-19 pandemic. However, evidence is accumulating that biocides can select for undesirable traits in bacterial pathogens, including antibiotic resistance. Despite this, the mechanisms underpinning changes in biocide susceptibility in important bacterial pathogens are not well understood. It is also unclear how biocide exposure and associated mutations impact clinically relevant microbiomes and biofilms. The aim of this study was to identify mechanisms involved in reduced chlorhexidine susceptibility in *Klebsiella pneumoniae*.

**Methods:**

A library of mini-Tn5 mutants was generated from the WT *K. pneumoniae* pre-antibiotic Murry isolate M109 and screened to identify those with reduced susceptibility to chlorhexidine (CHD). Whole genome sequencing and comparison to the WT genome were used to identify genes disrupted by Tn insertions and any spontaneous secondary mutations. Tn-mutants exhibiting at least a four-fold increase in CHD MBC and lacking any secondary point mutations were selected for further study and characterized to assess polar effects of Tn insertion, and evaluate a range of relevant phenotypes (growth, biofilm formation).

**Results:**

2 mutants out of 1584 mini-Tn5 *K. pneumoniae* M109 mutants screened exhibited a 4-fold increase in CHD MBC compared to the parental WT, with no secondary mutations. Tn insertions in these mutants were mapped to the promotor region of *RcnA* (encoding a nickel/cobalt efflux system), and the M109 plasmid upstream of *FecA* (encoding a TonB-dependent Fe(3) dicitrate receptor). Biofilm formation in these mutants was significantly increased in LB medium, but not artificial urine medium compared to the WT.

**Conclusions:**

These findings indicate the disruption in of *RcnA* and *FecA* can reduce CHD susceptibility and modulate biofilm formation in *K. pneumoniae* M109. However, changes in biofilm formation were only observed in LB media suggesting these may not be relevant to CAUTI.

